# Emerging applications of nanotechnology in context to immunology: A comprehensive review

**DOI:** 10.3389/fbioe.2022.1024871

**Published:** 2022-11-14

**Authors:** Hifsa Mobeen, Muhammad Safdar, Asma Fatima, Samia Afzal, Hassan Zaman, Zuhair Mehdi

**Affiliations:** ^1^ Department of Allied Health Sciences, Superior University, Lahore, Pakistan; ^2^ Centre of Excellence in Molecular Biology, University of the Punjab, Lahore, Pakistan; ^3^ Pakistan Institute of Quality Control, Superior University, Lahore, Pakistan; ^4^ Centre for Applied Molecular Biology, University of the Punjab, Lahore, Pakistan

**Keywords:** nanoscience, therapeutic nanoparticles, nano medicines, innate and adaptive immunity, nanomaterials nanoimmunostimulants

## Abstract

Numerous benefits of nanotechnology are available in many scientific domains. In this sense, nanoparticles serve as the fundamental foundation of nanotechnology. Recent developments in nanotechnology have demonstrated that nanoparticles have enormous promise for use in almost every field of life sciences. Nanoscience and nanotechnology use the distinctive characteristics of tiny nanoparticles (NPs) for various purposes in electronics, fabrics, cosmetics, biopharmaceutical industries, and medicines. The exclusive physical, chemical, and biological characteristics of nanoparticles prompt different immune responses in the body. Nanoparticles are believed to have strong potential for the development of advanced adjuvants, cytokines, vaccines, drugs, immunotherapies, and theranostic applications for the treatment of targeted bacterial, fungal, viral, and allergic diseases and removal of the tumor with minimal toxicity as compared to macro and microstructures. This review highlights the medical and non-medical applications with a detailed discussion on enhanced and targeted natural and acquired immunity against pathogens provoked by nanoparticles. The immunological aspects of the nanotechnology field are beyond the scope of this Review. However, we provide updated data that will explore novel theragnostic immunological applications of nanotechnology for better and immediate treatment.

## 1 Introduction

### 1.1 The emergence of nanosciences and nanotechnology

Nanoscience has emerged as an imperative discipline in the recent decade and can be defined as the science of nanomaterials (Ashraf et al., 2021). The term “Nano” originates from a Greek word meaning “Dwarf” referring to a very small-sized object. It is also used as a prefix in science meaning 1 nm is equal to 1 millionth of mm or 1 billionth of m (Boholm, 2016). The word “nanoscience” can be traced back to the era of Greeks and Democritus when the matter was considered a serious riddled point of concern to be debated whether it is continuous or not, to be broken into smaller and shatterproof particles, which are now termed by scientists as atoms (Bayda et al., 2019). The distinguishing line between nanoscience and nanotechnology lies in the fact that nanoscience explains the size, structure, and physical and chemical properties of nanomaterials (Jeevanandam et al., 2018) while nanotechnology is the practical application of the nanoscience for assembly, manipulation (Komal, 2021), control, and manufacturing of nanoscale material. Nanotechnology is a catch-all phrase for materials and devices that operate at the nanoscale (Hulla et al., 2015) and is considered the frontier of the 21st century in modern research (Ahmed et al., 2020).

Nanotechnology has gained importance in the engineering and application of nanomaterials from the fact that physiochemical and biological properties of materials vary remarkably at nanoscale dimensions from their properties at the normal stage (solid phase). The main key feature for such behavior lies in small size and increased surface area at the nanoscale and following the laws of quantum mechanics instead of classical physics (Barkalina et al., 2014). One striking example of such behavior is the catalytic property of gold due to increased surface area to volume ratio at the nanoscale which is chemically inert at the normal scale (Asha and Narain, 2020). Nanotechnology has found its applications in a wide range of disciplines from industries, buildings, military, and agriculture, to the medical sector (Filipe and Ferreira, 2021).

In the last decade, biomedical application of nanotechnology in drug delivery, tissue engineering, and diagnostics purpose has increased greatly improving the efficacy of these technologies. The rapid expansion of nanotechnology in healthcare can be estimated by the fact that the number of medical-related patents has dramatically increased from 200 per year in 2000–10,000 per year in 2010 with an increasing rate day by day (Barkalina, Charalambous, Jones, & Coward, 2014). Nanotechnology can play a part in the inflection of the immune system thereby paving the way for new therapies for widespread diseases like HIV, cancer, and diabetes. Nanocarriers can exert specific effects on immune cells due to their characteristic physiochemical properties and unique composition (Dacoba et al., 2017).

### 1.2 The building blocks of nanotechnology

Nanomaterials (NMs) are basic entities in nanotechnology and can be explicated as materials that are formed using principles and methodology of nanotechnology with length and diameter in the size range of 1–1,000 nm and 1–100 nm respectively (Boverhof et al., 2015). Many types of nanomaterials can be engineered such as nanoparticles, nanospheres, nanocapsules, and nanotubes (Chang, 2019). Precisely designed and functional nanostructures like, nucleic acids, tiny cell structures, and proteins can also be found in nature (Behari, 2010). Nanoparticles (NPS) can be grouped into various categories depending on origin, shape, size, structure, and properties (physical, chemical, electronical, mechanical, optical, and quantum). There are many types of nanoparticles like polymeric NPs, magnetic NPs, nanosuspensions, and nanocrystals. They are characterized by unique properties due to their small size and high surface area-to-volume ratio. They play a very important role in commercial and domestic applications such as; catalysis, imaging, and medical and environmental applications (Khan et al., 2019).

Nanospheres can be described as microscopic structures with a regular form of a sphere. It is composed of a solid polymeric matrix without any polymeric shell. They are used to deliver bioactive substances in the deeper layers of skin with enhanced penetration ability (Frank et al., 2019). Nanocapsule is a polymeric capsule of nanoscale size. It is a membrane-walled structure known as a polymeric shell with an oil core, containing drugs in it at the nanoscale level. They play a vital role in the delivery of the drug by enhancing drug-loading efficiency due to encapsulated oil core, protecting and releasing an accurate dose of the drug to the target (Deng et al., 2020).

There are different criterion-based classifications of nanomaterials based on;

1. Source/Origin
2. Morphology
3. Dimensions
4. Phase of Matter
5. Material
6. Synthesis
7. Toxicity

## 2 Origin/source based-classification

Nanomaterials can be categorized into three main types natural, engineered/artificial, and incidental nanomaterials depending on the source.

Natural nanomaterials can be described as a material produced by biogeochemical processes naturally without any involvement of anthropogenic activity. Naturally occurring nanomaterials cover all the earth’s spheres such as; the atmosphere (gasses), hydrosphere (oceans, lakes, and rivers), lithosphere (rocks), and biosphere (living organisms) (Hochella et al., 2015; Sharma et al., 2015).

Nanomaterials such as; nanoparticles and nanostructures can be seen in all simple (microorganisms) and complex living organisms (humans, animals, and plants). Advanced nanotechnology has contributed a lot to the understanding and utilization of organisms for beneficial biological and medical applications due to the presence of nanomaterials. Nanostructures are the keystone for life in the world. Animals, plants, and insects use nanomaterials for their survival and protection from harsh environments. Human beings contain nanostructures in the genetic material, bones, blood, and cartilage that maintain proper anatomy and physiology (Jeevanandam et al., 2018).

Engineered/artificial nanomaterials have been successfully designed and developed with desired properties and applications in different areas of interest by human beings. They synthesize them intentionally by using different physical, chemical, and biological techniques (Wagner et al., 2014). Anthropogenic activities such as; cooking and fuel exhaustion (Linak et al., 2000) welding, and smelting (Mandal and Ray Banerjee, 2020) are also playing a key role in the manufacturing of synthetic nanomaterials like carbon nanoparticles (De Volder et al., 2013) and Titanium dioxide nanoparticles (Weir et al., 2012) in cosmetic products and Sun blockers.

Incidental nanomaterials or waste particles come into existence accidentally and unintentionally due to direct or indirect anthropogenic activity. They are generated as a byproduct of industrial and natural processes such as; the emission of fuels from transport vehicles, welding, combustion processes, forest fires, Photochemical reactions, volcanic venting, and sloughing off of skin, hair, and leaves from animals and plants respectively. They are highly toxic and affect the quality of airways, waterways, and groundways. Anthropogenic activities are playing the least role in the production of atmospheric aerosols which is only about 10% of total aerosol as compared to naturally produced aerosols (Taylor, 2002).

## 3 Design-based classification

**FIGURE 1:**
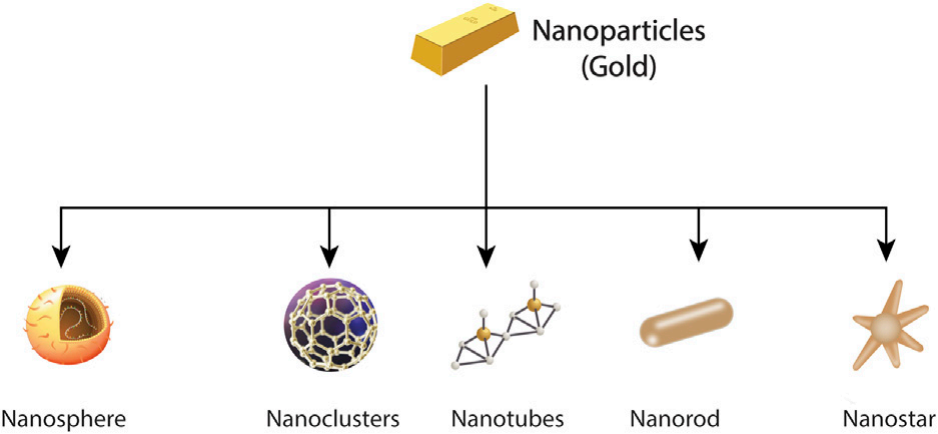
Design-based classifications of gold nanoparticles.

Nanomaterials can be seen in different forms like nanospheres, nanocages, nanoclusters nano reefs, nanotubes, nanorods, nanowires, nanostars, nanoshells, nano prisms, *etc.* For example, different shapes of gold nanoparticles (Landvik et al., 2018). Different shapes of gold nanoparticles are shown in Figure 1.

## 4 Dimension-based classification

**FIGURE 2:**
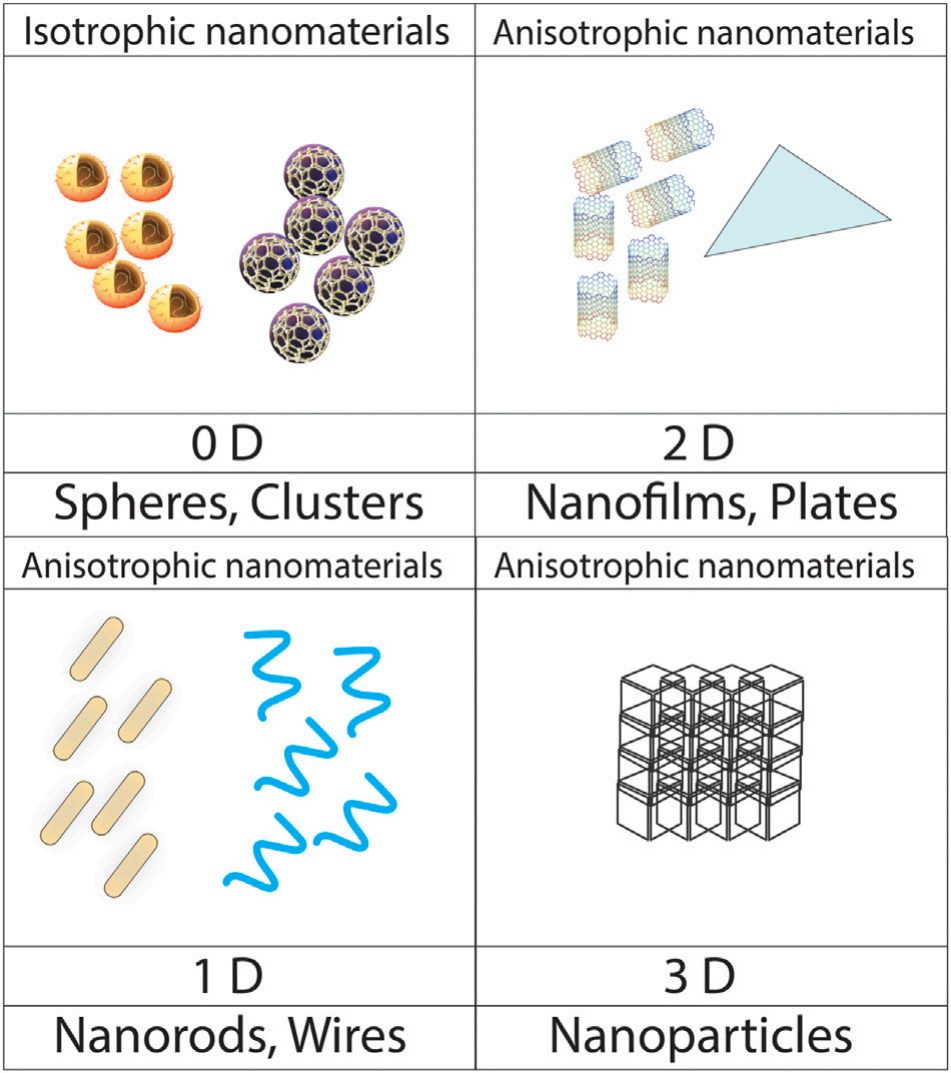
Dimension-based classification of nanoparticles.

The nanomaterials can be grouped into four types based on dimensions such as; bulk nanomaterials, nanoplates, nanotubes, and nanospheres with three, two, one, and zero dimensions respectively. This dimension-based classification depends on the movement of electrons along the dimension of the x, y, and *z* axis in nanoparticles as shown in Figure 2.

Bulk nanomaterials can be observed with three-dimensional (3D) structures in the nanoscale range. This class consists of diamonds, graphite, polycrystals, *etc.* The movement of electrons takes place along the x, y, and *z*-axis. They are widely used in biomedical sciences as a catalyst. Nanoplates are thin layered structures with nanoscale sizes in which electrons move along the *x* and *y*-axis. Carbon-coated nanoplates and Graphene sheets are two-dimensional (2D) structures. They are considered building blocks of nanodevices (Jibowu, 2016).

The one-dimensional (1D), thin film-sized nanomaterials are found to be only one dimension at the nanoscale with a wide range of applications in chemistry, electronics, and pharmaceutics (Gopi et al., 2016). Carbon nanotubes (CNTs), Gold nanowires, Nanoribbons, and nanorods are one-dimensional nanomaterials in which electrons move along the *x*-axis (Hangarter et al., 2010). Nanospheres are zero-dimensional nanostructures with all dimensions in the nanoscale range. They consist of quantum dots, Fullerenes, and Gold nanoparticles with the restricted movement of electrons in all directions (Pokropivny and Skorokhod, 2007; Lee et al., 2015).

## 5 The phase of matter based-classification

The four different types of nanomaterials based on the phase of matter are nanocomposites, nanofoams, nanoporous and nanocrystalline. Nanocomposites are solid forms of nanomaterials with at least one nanoscale dimension. They are playing role in pharmaceutical and therapeutic applications.

Nanofoams consist of two phases at the same time with at least one phase in the nanoscale dimension. They may be liquid or solid forms of nanomaterials filled with gases. Carbon nanofoamsare used in magnetic resonance imaging by being injected into blood vessels to increase the quality of the image.

Nanoporous is a solid form of material with a porous structure with nanoscale dimension. They are used in drug delivery, gas purification, and energy storage devices. Nanocrystal is a polycrystalline material containing crystals with at least one nanoscale dimension (10^9^ m). They are used to treat wounds or burns as an antibacterial and anti-inflammatory agent (Landvik et al., 2018).

## 6 Material-based classification

Nanomaterials can be categorized into four types based on material such, Carbon-based nanomaterials, Inorganic-based Nanomaterials, Organic-based nanomaterials, and Composite-based nanomaterials.

Carbon-based nanomaterials: These nanomaterials are composed of carbon and are found in Fullerenes (C60), carbon nanotubes (CNTs), carbon nanofibers, and graphene. Different techniques are used to manufacture these materials such as; Laser ablation, Arc discharge, and chemical vapor deposition (CVD) (Patel et al., 2019). These carbon-based nanomaterials have a wide range of applications in different fields of medicine and the environment as antimicrobial agents, and environmental sensors (Mauter and Elimelech, 2008).

Inorganic-based nanomaterials: These nanomaterials are highly stable as compared to organic-based nanomaterials with substances like metals, metal oxides, and meta salt. They can be manufactured into metals and metal oxides such as; Au or Ag NPs and TiO2 and ZnO NPs respectively. They contain an inorganic central core with fluorescent, magnetic, electronic, and optical properties. These non-toxic and biologically compatible nanoparticles do not contain carbon. Different Inorganic-based nanomaterials such as; quantum dots (QDs), gold nanoparticles (AuNPs), silver nanoparticles (AgNPs), and carbon nanotubes (CNTs) have a broad range of applications (Collier et al., 2017).

Organic-based nanomaterials: These are two-dimensional nanosized materials showing unique physical and chemical properties due to their typical shape and size. The organic-based nanomaterials are ferritin, liposomes, micelles, peptide-based, and dendrimers. They are made of organic matter with noncovalent interactions for self-assembly (Khalid et al., 2020). These are non-toxic and Eco-friendly nanoparticles with variable morphology such as; hollow spheres (Liposomes) and nanocapsules with Temperature and light-sensitive properties (Tiwari et al., 2008). These polymeric nanoparticles are considered the ultimate choice for therapeutic drug delivery to combat disease (Mansha et al., 2017).

Composite-based nanomaterials: Composite-based nanomaterials consist of two or more components of a nanoscale range with specific physical and chemical properties such as; metalorganic frameworks. The composites are composed of different combinations of carbon-based, metal-based, and organic-based nanomaterials and any form of metal, ceramic, or polymer bulk materials. Their advanced functions and properties depend on the size, composition, and atomic order of aggregates. They are scientifically and technologically advanced materials and play a crucial role in different applications such as; efficient catalysts, metal-semiconductor junctions, modifiers of polymeric film for packaging, and optical sensors (Tanjina Hasnat, 2021).

### 6.1 Synthesis-based classification

The two broadly divided synthetic methods through which nanoparticles can be made for different purposes are; Top-down synthesis and Bottom-up synthesis.

### 6.2 Top-down synthesis

**FIGURE 3:**
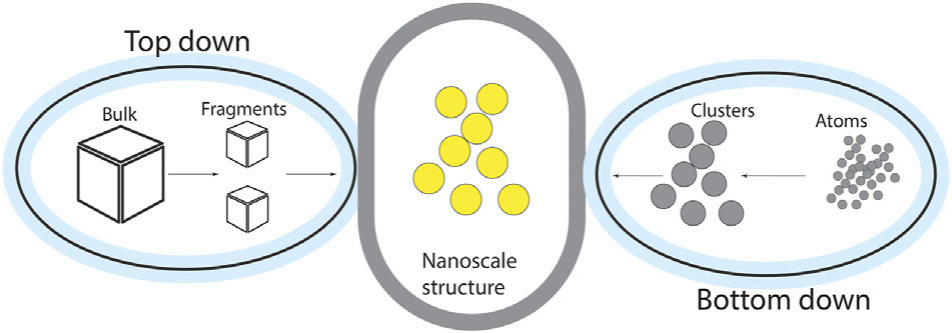
An overview of Top-down and bottom-up approaches.

It is also known as a destructive method in which larger bulk materials are broken down into smaller molecules which are ultimately converted into nanoparticles. This is intrinsically a simpler approach to designing structures with desired properties. The imperfection of surface structure, uncontrolled size, and structure of nanoparticles are serious issues faced by experts. In this technique, nanowires made by lithography are not smooth and possess structural defects on their surface. High-energy wet ball milling, electron beam lithography, atomic force manipulation, gas-phase condensation, aerosol spray, and etching are examples of this approach (Iravani, 2011). An overview of this approach is shown in Figure 3.

### 6.3 Bottom-up approach

The bottom-up approach is Eco-friendly and economical, which constructs material from the bottom with less production of waste. Sol-gel synthesis (Ramesh, 2013), colloidal precipitation, hydrothermal synthesis, electrodeposition, etc, are bottom-up techniques (Figure 3), used for nanostructures and nanomaterials preparation. The bottom-up approach is considered more ideal as compared to the Top-down approach in the preparation of nanoparticles due to fewer defects and homogeneity.

### 6.4 Biological method

Biological sources including plant extract, fungi, and bacteria are used for the synthesis of nanoparticles. This method is more preferred, Eco-friendly, simple, and economical as compared to other physical and chemical methods (Iravani, 2014).

## 7 Toxicity-based classification

**FIGURE 4:**
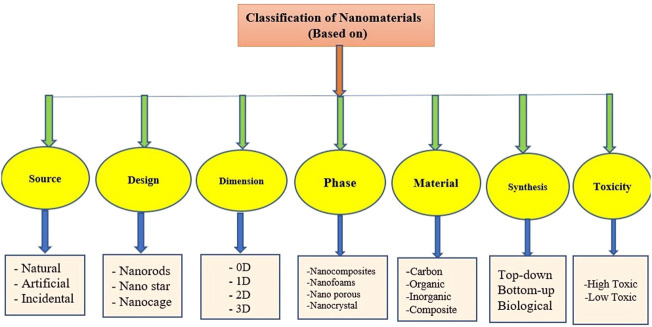
Schematic diagram showing classifications of nanoparticles.

Nanomaterials are mostly used for beneficial purposes to facilitate human beings but they are also a factor in causing diseases by releasing toxic ions. They are categorized into three categories based on solubility and toxicity as shown in Figure 4. (I) Highly soluble nanoparticles affect the lungs and other viscera by releasing toxic ions such as; zinc oxide (ZnO). (II) Poorly soluble low-toxicity nanoparticles cause less toxicity as compared to highly soluble nanoparticles. They cause Fibrosis and cancer by the release of Titanium oxide (TiO). (III) Poorly soluble highly toxic nanoparticles cause Fibrosis and cancer by generating reactive oxygen species (ROS) such as; Nickel oxide (NiO) (Kuempel et al., 2012).

## 8 General applications

Nanotechnology has improved and revolutionized all fields of the world like healthcare, agriculture, military, aerospace, electronics, textile, sports, cosmetics, environmental preservation, and air purification.

### 8.1 Nanomedicine

Nanomedicine is the medical application of nanotechnology, playing a key role in diagnostic and therapeutic purposes efficiently and effectively. Nanopharmaceuticals utilize nanomaterials for target drug delivery, antibacterial activity, and as nano biosensors. These nanoparticles have several other applications in nanomedicine such as;

• Drugs are successfully targeted with optimal concentration and extended-release (ER) dosage for therapeutic purposes (Pradhan et al., 2021).
• Gene therapy with polymeric nanoparticles can be delivered to the desired target effectively and economically with low toxicity and protracted stability (Rai et al., 2019).
• Nanomaterials such as; nanoarrays, nanowires, and nanotubes are widely used for the prompt detection of biomarkers associated with various diseases with low sample consumption. It is considered an emerging, sensitive and successful technology for the detection of bacteria, viruses, and cancer cells at an early stage (Mocan et al., 2017).
• Some CuO NPs are used in the noninvasive methodology-based theranostic process because they possess a contrast-enhancing effect in Magnetic resonance imaging and ultrasound (Aresteanu et al., 2020; Zeng et al., 2020).

### 8.2 Nanoelectronics

Nanoelectronics is an advanced technology that is used to increase the potential of electronic devices and memory chips while reducing their weight, size, and power consumption.

• Electrodes manufactured from nanowires enable flat panel displays to be flexible and thinner.
• Nanolithography is used for the fabrication of chips.
• The transistors are made of nanowires, assembled on glass or thin films of flexible plastic.
• Magnetic Random Access Memory (MRAM) enabled by nanometer-scale magnetic tunnel junctions saves even encrypted data efficiently and effectively during a system shutdown or crash.
• Nanostructured polymer films such as; organic light-emitting diodes (OLEDs) are incorporated into the Display screens of TVs, laptop computers, and mobile phones which offer brighter and wider images with low power consumption (Subramanian and Lee, 2012).

### 8.3 Nanoagriculture

Nanotechnology has played a vital role in the agricultural industry with the development of nano-fertilizers, nano pesticides, and plant gene transformations to produce plants with desirable characteristics. With the increasing global population, it is necessary to make advancements in farming methods to generate higher yields with minimal waste production to meet the rising food demand by using nanotechnology techniques.

• Nano-fertilizers such as; Amorphous Calcium Phosphate (ACP) nanoparticles is economical and eco-friendly preparation used to increase crop yield (Carmona et al., 2021).
• Natural and synthetic resources are used to improve the quality and quantity of crops to meet the rising demands of food with an alarming and unchecked increase in the global population.
• Seed germination of tomato, wheat, and maize is positively stimulated with the application of multi-walled carbon nanotubes (MWCNTs) (Srivastava and Rao, 2014).
• The diagnostic and treatment purposes of different plant diseases have become possible now with the development of Nano sensors (Shang et al., 2019).

### 8.4 Nanotechnology in military

Nanoparticles found in the material of soldiers’ uniforms make it more durable and protect soldiers from many dangers such as harsh environmental conditions and chemicals.

• Military Uniforms are manufactured with durable nonwoven light weight and breathable fabrics which possess different properties such as; water-repellent and heat resistance.
• Military equipment and weapons have become more advanced with the utilization of nanotechnology (Glenn, 2006).
• Other applications of nanotechnology in the military such as; Water and Bullet proof vests, Lifesaver bottles, Soldier smart cards, and Smart helmets are also helpful for soldiers in different ways.
• Wound dressings coated with nanoparticles allow the controlled release of drugs and growth factors for wound healing in the predetermined period.
• Chemical and biological nano sensors are used for the detection of harmful weapons (Ramsden, 2012).

### 8.5 Nano fabrics

Nanotechnology is widely used in the development of textiles with desired characteristics, such as durability, wrinkle-free, soft hand, water, heat, and stain repellence, and antimicrobial properties.

• Water, heat, and stain-repellent property of a fabric is due to nano-whiskers, which are added to the fabric to create a peach fuzz effect.
• Nano fabrics protect the wearer from the weather and ultraviolet rays by blocking their direct transmission to the skin through fabrics.
• Synthetic fibers such as nylon and polyester possess a charge after absorption of water but Cellulosic fibers do not carry any charge due to the presence of moisture content. Nano-sized titanium dioxide (TiO_2)_ and zinc oxide (ZnO) whiskers impart anti-static properties to synthetic fibers.
• Nano-sized silver, titanium dioxide, and zinc oxide are used as antimicrobial agents in nano fabrics (Saleem and Zaidi, 2020).

### 8.6 Nanotechnology and cosmetics

Nanotechnology has contributed a lot to the protection of skin from ultraviolet rays and reversing the aging process at the cellular level through nanospheres, nano-emulsions, and nanoparticles. They alter the properties of cosmetic products including color, transparency, solubility, and chemical reactivity. Nanotechnology applications in cosmetics and skin care include:

• Zinc oxide and titanium dioxide appear transparent at the nanoscale rather than opaque, allowing their utilization in Sun blockers, foundations, and moisturizers (Bilal and Iqbal, 2020). Aluminum oxide nanoparticles used in foundations and face powders facilitate with ‘‘soft-focus’’ effect to conceal visible wrinkles. Carbon ‘‘fullerene’‘. Nanoparticles are also used in anti-aging creams and lotions. Therefore, the cosmetics industry has revolutionized itself by using nano-ingredients in the characterization of cosmetics (Singh and Nanda, 2012).
• Nano emulsions are synthesized by encapsulating nutrients in nanoparticles suspended in a liquid. They are found in Sun blockers, anti-wrinkle creams, foundatins, face powders, soaps, shampoos, and conditioners (Özgün, 2013), which penetrate the skin deeply to deliver nutrients to the *epidermis* and dermis (Chevalier and Bolzinger, 2019).
• →Nanogold is an antiaging, antioxidant, antibacterial, antifungal, and anti-inflammatory agent, which is used in cosmetic products to protect skin from free radicals (Yeh et al., 2012). Nanogold exhibit various properties and shapes such as nanoshells, nanospheres, nanostars, nanocubes, nanorods, and nanoclusters which affect the resonance frequency of gold nanoparticles. The color of nanogold also changes from red—purple—blue—virtually black due to aggregation (Khan et al., 2014).

### 8.7 Nanobiotechnology

Nanobiotechnology has manufactured devices and systems at the nanoscale level to develop biological, diagnostic, and therapeutic assays to achieve targets in nanomedicine, molecular nanotechnology, food microbiology, and tissue engineering. Nanobiotechnology is used for various purposes such as;

• Nanofluidic biochips such as; lab-on-chip are used to perform biomedical analysis of liquids with very minute quantities and the detection of macromolecules such as DNA and proteins (Kovarik and Jacobson, 2009).
• Chip-based biosensors are also known as Point-of-care biosensors, which are used for the diagnosis of different infectious diseases and the detection of biomarkers precisely with a very small volume of samples (McRae et al., 2016).
• Solid-state nanopore sensors are tiny holes of proteins, which are used for direct and real-time analysis of DNA sequencing and RNA fragments at the single molecule level (Xue et al., 2020). Some carbon nanomaterials like carbon nanotubes, carbon nanofibrils, and graphite used in optoacoustic transducers improve their optoacoustic conversion efficiency and have laser damage threshold (Li et al., 2022).
• Nanomaterials are composed of nanoparticles (NPs) such as; magnetic NPs, AuNPs, AgNPs, silica NPs, quantum dots (QDs), polymeric micelles, liposomes, dendrimers, and fullerenes are used in biosensing, bioimaging, diagnostic, and therapeutic applications to facilitate healthcare industry economically (Nagamune, 2017).
• For efficient, highly-selective phosphate removal from wastewater, a new lanthanum carbonate-grafted ZSM-5 zeolite (LC-ZSM-5) was created (Yang et al., 2021).

### 8.8 Nano-oncology

Cancer is the second leading cause of death worldwide after cardiovascular diseases (CVS) (Luisa and Valentina, 2021). It is characterized by abnormal division of cells which even affects the normal healthy cells (Tran et al., 2017). Thus, it is necessary to develop an efficient technique for the early-stage diagnosis and treatment of cancer.

Nanoparticles (NPs) are widely used in anticancer therapy to deliver chemotherapeutic drugs to the target site (Dong et al., 2019; Zhang et al., 2019) The stealth properties of NPs can be improved by covering them with chemical or biological coatings, which ultimately reduce the formation of aggregates in the body fluids. The maximum delivery of chemotherapeutic agents specifically to the target site can be achieved through conjugation with targeting ligands (Limongi et al., 2019).

The increased permeability and retention effect (EPR) enable NPS to accumulate into cancers because they can cross the tumor vasculature easily and poor lymphatic drainage retains them to facilitate their therapeutic effect (Youn and Bae, 2018). A supramolecule is developed by grafting the epitopes of bioactive D-peptide onto the mini protein template. This functions as a p53 antagonist and possesses An advantageous pharmacological profile that includes endosome escape, intracellular reductive response, passive tumor targeting, and cell membrane penetration (Yan et al., 2020). An antitumor Benzofuro had been synthesized by using Nanopalladium as a catalyst. It showed promising results against T-24 and Hela cell lines (Wang et al., 2019).

Chemotherapeutic agents react strongly with abnormally dividing cells and inhibit the cell division process by interfering with DNA synthesis. These agents also damage normal healthy cells along with the treatment of cancer. Advanced nanotechnology-based therapeutic drug delivery system is more effective in treating cancerous cells with a chemotherapeutic agent with minimal effect on non-tumor healthy cells in the body by controlling their rate of release on the target site in specified time duration. The polymer (poly lactic-co-glycolic acid) (PLGA) is hydrophilic and used as a nanocarrier. It remains in the blood circulation longer enough without being used and removed by the liver metabolism (Jibowu, 2016).

### 8.9 Green nanotechnology

A branch of green technology that utilizes plant products for the protection of the environment by reducing greenhouse gases, pollution, and hazardous waste as well as by saving the ozone layer, water, energy, and non-renewable raw materials (Verma et al., 2019).

Green Nanotechnology has improved the environment in many ways by producing efficient and economical energy with generating less pollution but sometimes it may be undesirable when it affects the food web (Almeida et al., 2020).

• Solar cells have become more efficient, economical, and smaller in size with the advancement of nanotechnology. They use solar energy, which is a renewable resource (Banin et al., 2020).
• Nanotechnology has played role in the treatment of groundwater, surface water, and wastewater by reverse osmosis and nanofiltration, contaminated by microorganisms, organic and inorganic solutes, and toxic metal ions, microorganisms (Jain et al., 2021).
• Nano remediation of soil and sediment with nanoparticles has reduced pollution in soil caused by industrial activities (Baragaño et al., 2020).
• Nanoparticles are efficiently playing role in the removal of toxic materials from gases such as; CO and SO_2_ (Guerra et al., 2018).
• Nanotechnology has been proven to be a beneficial tool in the cleaning up of oil spills in the aquatic system to save aquatic biodiversity by applying magnetic nanomaterials such as functionalized super-paramagnetic iron oxide nanoparticles (SPIONs) and magnetic carbon nanotubes (CNTs). Nanowires are used to clean up oil spills by absorbing them up to twenty times their weight in hydrophobic liquids and rejecting water due to the coating of the being water-repellent (Singh et al., 2020).
• Nanotechnology has been considered a successful tool to improve the quality of air by using nanoparticles. They transform toxic gases (CO, NO, and hydrocarbons) of industrial factories into harmless ones by sensing, detecting, and remediating them (Mohamed, 2017).

### 8.10 Nanotechnology in immunology

Nanotechnology has been proven to be a promising approach for successful applications in the field of immunology with the production of nano vaccines, adjuvants, anticancer drugs, and immuno-modulatory cytokines to combat infectious and autoimmune diseases.

Nanoparticles possess the strong potential to stimulate immunity due to their unique properties. They act as adjuvants by protecting, stabilizing, and presenting foreign particles (antigens) to antigen-presenting cells (APC) like macrophages, dendritic cells, and B cells with controlled release of antigen in blood circulation and surrounding tissue for effective, prolonged, and strong immune response (Lenders et al., 2020).

The properties of nanoparticles can be a “double-edged sword,” as they exhibit immunotoxic and immunomodulatory functions. Metal-based nanoparticles have various effects on different cells such as;

• Interaction with cells possessing Toll-Like receptor signaling (TLR) and antigen presentation properties like macrophages and dendritic cells for the expression of proinflammatory cytokines and activation of T cells.
• Interact with innate immune cells like neutrophils, mast cells, and NK cells for activation and augmentation of innate immunity.
• Interaction with acquired immunity-related T and B cells to combat viral and bacterial infections (Luo et al., 2015).

#### 8.10.1 Immunomodulation with nanoparticles

Layered defenses of increasing specificity protect us from infections. Physical barriers, skin, mucosal membrane of the mouth, respiratory, gastrointestinal, and urogenital tract limit the entry of microbes (bacteria, fungi, and parasites) and viruses which are non-specific innate immunity that provides an immediate response. These mucosal membranes are selectively permeable for nutrients, water, ions, and gases while limiting the entry of pathogens upon exposure (Nel et al., 2021).

The immune system has been divided into two major types: innate immunity and adaptive immunity. Innate immunity is the first line of the defense system, which depends on pattern recognition receptors (PRPs) to recognize conserved pathogen-associated molecular patterns found on pathogens (PAMPs). Therefore, innate immunity plays a key role in the early recognition and subsequent basic microbial eradication by phagocytes (macrophages and neutrophils) and their cytokines through inflammatory processes (Amarante-Mendes et al., 2018; Nickoloff, 2019).

When innate immunity fails to eradicate pathogens, adaptive immunity or specific immunity responds *via* humoral immunity and cell-mediated immunity but it starts after a lag time (Vincenzo et al., 2015). Antigen-presenting cells (APC) such as dendritic cells, macrophages, and B cells present antigens in association with class II MHC to CD4 helper T cells. Then CD4 cells respond to APC by producing cytokines while CD8 cells respond by apoptosis and cytokines (Gaudino and Kumar, 2019).

IFN-gamma produced by CD4 cells enhances the microbicidal activity of macrophages. CD4 cells produce IL-2 and enhance the cytotoxic action of CD8 cells. CD4 cells also help B cells in the production of antibodies by releasing IL-4 and IL-5 (Cruz-Adalia et al., 2017).

The immune system perceives nanoparticles as foreign substances and eradicates them from the body immediately. However, if these foreign nanoparticles are not considered a threat-causing factor, they are completely tolerated by the immune system without causing any harmful effects. Therefore, it is the point of concern to analyze the response of the body’s immune system with the development of a novel nanoparticle for *in vivo* applications such as gene or drug delivery to minimize undesirable consequences (Muhammad et al., 2020).

Nanoparticles have been designed for safe and targeted drug delivery, vaccination, and tumor therapy without any immunological harmful consequences. Three major harmful consequences related to the immune system of the body must be considered with the development of a novel nanoparticle for *in vivo* application.

• The first consequence is immunostimulant which is related to the destruction or rejection of nanoparticles with eradication from the body by the defensive immune system.
• The second is immunotoxicity, which could affect the functioning of the local and systemic immune system due to exposure to toxins and cause pathological problems.
• The third is immunocompatibility, in which the immune system could not be interfered with by any foreign object (Dobrovolskaia et al., 2016).

Several techniques can be used to load drugs with the nanoparticle for targeted and efficient drug delivery such as entrapped drug inside the nanoparticle (encapsulation), coated drug on the surface of the nanoparticle (coating), and chemically linked with the nanoparticle, which helps them to evade the phagocytic cells of the immune system. The unique properties of nanoparticles such as size, charge, hydrophobicity, and hydrophilicity direct nanoparticle-coated drug to the target safely (Dong et al., 2019).

Nanoparticles developed by encapsulation with polyethylene glycol (PEG) or PEGylation are widely used in targeted drug delivery and nanotechnology due to “stealth” properties and biocompatibility. PEGylation plays a vital role in drug delivery systems by evading or shielding them from recognition of the immune system (Suk et al., 2016). However, sometimes the immune system produces PEG-specific antibodies after the introduction of PEG-coated liposomes (Mohamed et al., 2019). Some important nanoparticles with their applications in immunotherapy are listed in Table 1.

**TABLE 1:** Nanoparticles uses in immunotherapy.

Nanoparticle type	Size (nm)	Applications	Target	References
ZnO NPs	30–150	Vaccine Carrier	TLRs	Sharma et al. (2019)
Polymeric NPs (PLGA)	100–200	Vaccine Carrier, adjuvant	DCs	Kim et al. (2018)
Exosome	40–100	Vaccine Carrier	T Cells	Romagnoli et al. (2015)
Liposome	100–160	Vaccine Carrier/Adjuvant	DCs/macrophage	Koshy et al. (2017)
Metal NPs (AuNPs)	20–80	Vaccine Carrier/Adjuvant	DCs	Almeida et al. (2014)
Metal NPs (IONPs)	20–80	Antibody carrier	DCs	Choi et al. (2015)
Polymeric NPs (Micellar)	25–50	Vaccine Carrier	DCs	Jeanbart et al. (2015)

#### 8.10.2 Impact of nanoparticles on the stimulation of innate immunity

Innate immunity or natural immunity is the first line of defense of the body’s immune system which response non-specifically and immediately to encounter pathogens with the activation of pre-existing defensive mechanisms including physical, anatomical, chemical, and biological barriers. The main components of natural immunity are;

• physical barriers including skin, mucous membranes, and mucus.
• Anatomical barriers consist of phagocytes (polymorphonuclear leukocytes, monocyte, macrophages, and dendritic cells), basophils, eosinophils, mast cells, and natural killer cells (NK).
• Phagocytic cell enzymes include lysozymes, elastase, and protease.
• Circulating plasma proteins comprise pathogen recognition receptors (PRRs). Mannose-binding lectins (MBL), C-reactive proteins (CRP), and the complement system.
• Antimicrobial peptides such as; cathelicidin and defensins.
• Cell receptors like Toll-like receptors (TLR), Mannose receptors (MBL), and Dectins (Zhang et al., 2021).

**FIGURE 5:**
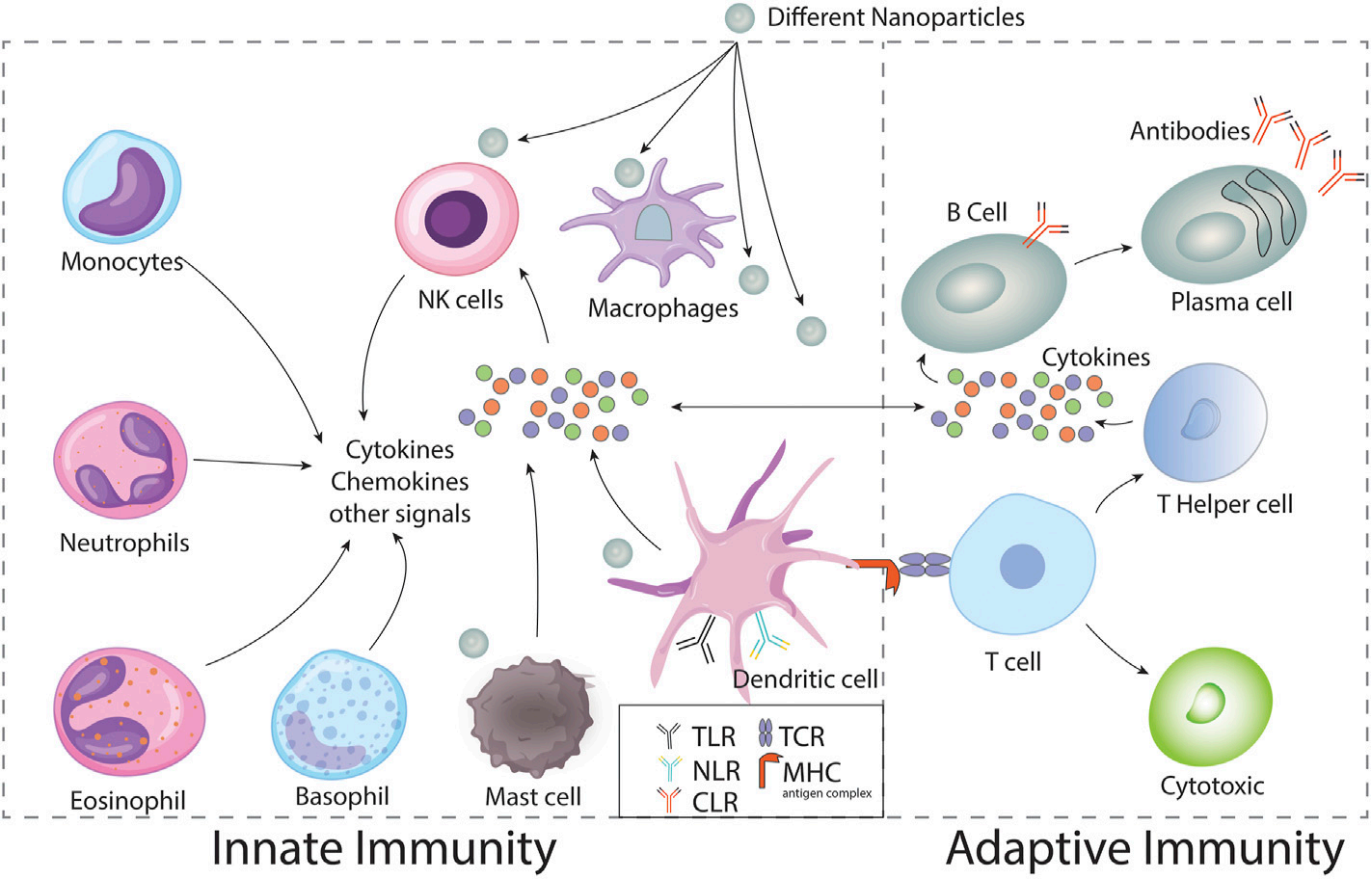
Nanoparticles from different sources Modulate innate and adaptive immune system.

In this review, we will first focus on the discussion of the enhanced stimulatory mechanism of cells related to innate immunity to resolve infections due to nanoparticle conjugation and then describe the impact of these nanoparticles on the cells of adaptive immunity with advanced specificity (Figure 5).

#### 8.10.3 Role of nanoparticles in toll-like receptor signaling

Innate immunity depends on the recognition of pathogen-associated molecular pattern (PAMPs) by pattern recognition receptors (PRRs), which belongs to the family of toll-like receptors (TLRs). Toll gene controlling the dorso-ventricular polarization of embryo, originally discovered in *Drosophila*, was found to be playing role in immunity against fungal infections in the adult fly. Toll-like receptors found on the surface of the cell are TLR1, TLR2, TLR4, TLR5, TLR6, and TLR10 while TLR3, TLR7, TLR8, TLR9, TLR12, and TLR13 are located in intracellular vesicles such as endosomes and lysosomes (El-Zayat et al., 2019).

• The activations of the TLR signaling mechanism stimulate macrophage and natural killer (NK) cells associated with phagocytosis and cytotoxic activity respectively.
• They increase antigen presentation *via* upregulating the expression of major histocompatibility complex MHC) and costimulatory molecules (CD80 and CD86) on antigen-presenting cells like dendritic cells, macrophages, and B-cells, leading to the activation of adaptive immunity.
• TLR agonists or nanoparticles are considered to be powerful adjuvants. TLR antagonists or inhibitors have been proven to be a therapeutic tool to treat septic shock and autoimmune diseases like systemic lupus erythematosus (SLE) and rheumatoid arthritis by downregulating the inflammatory responses (Gao et al., 2017).
• Nanoparticles like Titanium dioxide (TiO2) elicit an inflammatory response with the production of reactive oxygen species by increasing the expression of TLR7 on macrophages (Huang et al., 2017).
• ZnO nanoparticles possess anti-inflammatory and anti-microbial properties by the induction of MyD88-dependent proinflammatory cytokines through the signaling pathway of TLR (Moratin et al., 2021).

These advanced potential applications of nanoparticles may open novel and innovative directions for the synthesis and characterization of nanoparticle conjugates to meet advanced requirements in immunology.

#### 8.10.4 Immunostimulatory effect of nanoparticles on cytokine production

Cytokines are proteins that act as signaling molecules in mediating and regulating inflammatory protective mechanisms in innate and adaptive immunity. Inflammatory cytokines, such as IL-1, IL-6, and tumor necrosis factor- (TNF-α), stimulate inflammatory cells (neutrophils and macrophages), increase vascular permeability, and cause swelling and erythema. IL-8 is a chemokine that plays a key role in the recruitment of inflammatory cells to the targeted inflammatory sites after activation. Activated lymphocytes secrete Interferon- γ (IFN-γ) which is a primary activator and recruiter of macrophages to the site of infection (Kany et al., 2019).

NPS can trigger an immune response with the production of cytokines to resolve infection due to their particular physiochemical properties. The measured levels of these triggered cytokines act as biomarkers of nanoparticle immunomodulatory effects. TiO2 nanoparticles and nanodiamonds stimulate the production of the proinflammatory cytokine, maturation of dendritic cells, and activation of lymphocytes. ZnO nanoparticles play an important role in enhancing the expression of IFN-γ in lymphocytes and IL-12 in monocytes (Swartzwelter et al., 2020).

The more understanding we have of cytokine production triggered by nanoparticles, the better we can use the level of cytokines as biomarkers of the immunostimulatory properties of nanoparticles.

#### 8.10.5 Effects of nanoparticles on the cells of innate immunity

The cells of innate immunity are Natural Killers (NK), neutrophils, Macrophages, dendritic cells (DCs), mast cells, eosinophils, basophils, and gamma/delta T cells. We will discuss the effects of nanoparticles on these cells and utilize them for therapeutic purposes. Moreover, we will demonstrate the modulation of eosinophils, basophils, and gamma/delta cells with nanoparticles by reviewing the most recent data, which is still a challenging task.

#### 8.10.6 Augmentation of natural killer cell-based immunotherapy

NK cells are large granular lymphocytes of innate immunity, which do not require thymus for maturation. They play important role in defending the body against viruses naturally, as they do not require any prior exposure (immunologic memory) to that particular viral antigen, hence named “natural” killer cells (Bagheri et al., 2021).

NK cells regulate macrophages and T cells by interacting and activating them with the production of gamma interferon to kill phagocytosed bacteria. They also kill tumor and virus-infected cells non-specifically by the secretion of cytokines, Fas-Fas ligand-mediated apoptosis, and antibody-dependent cellular cytotoxicity (ADCC) Therefore, NK cells are considered a double-edged sword either to limit or worsen the situation of immune responses. It has been reported that patients with deficient NK cells can predispose to life-threatening infections. Hence, NK cell-based immunotherapy could be proved to be an effective strategy against tumors (Liu et al., 2021).

However, NK cell-based immunotherapy is still facing many challenges in treating cancer patients. The tumor microenvironment (TME) with altered immunogenicity disguise NK cells infiltration to the target (Nayyar et al., 2019). Thus, many strategies have been proposed for the expansion, activation, and infiltration of NK cells to the targeted tumor site (Cerwenka and Lanier, 2016; Fehniger and Cooper, 2016).

Recently, it has been reported that nanoparticles with multifunctional properties play a significant therapeutic role to treat various types of tumors with the augmentation of NK cells (Nam et al., 2019; de Lázaro and Mooney, 2020). The multifunctional properties of nanoparticles such as targeted cytokine delivery, tracking, identification, and advanced treatment of cancer have been proven to overcome challenges related to NK cell-based immunotherapy of cancer (Irvine and Dane, 2020; Phung et al., 2020).

The tracking and identification of cells can be made possible with the quantum dots (QD) imaging technique by labeling NK-92 MI cells with anti-CD56 antibody-coated QD705, a quantum dot that emits light in the near about infrared region. The NK-92MI injections were performed and tracked for 12 days after intra-tumoral injections. A significant reduction was observed in the size of the tumor with minimal toxicity after treatment with NK cells as compared to the control.

Quantum dots are used for imaging technology due to several advantages such as; high photostability, Narrow and symmetric peak of emission spectra, high quantum yield, long shelf life, color availability, and small size.

Some compounds cannot be used therapeutically and diagnostically due to the toxic and non-degradable nature of cadmium cores. So, it is highly needed to develop biodegradable and cadmium-free QDs for safe clinical applications (Shapovalova et al., 2018).

The updated data of this review will provide an alternative clinical treatment approach to cure and remove tumors with minimal toxicity of nanoparticles. It will also suggest innovative diagnostic imaging techniques with advanced infiltration of immunology to the three-dimensional (3D) target site without a surgical incision.

#### 8.10.7 Neutrophil-based delivery of nanotherapeutics

Polymorphonuclear neutrophils are considered a key component of innate immunity. They are the first ones fromthe leukocyte family that transmigrates to the target site during acute inflammation and release several types of proinflammatory mediators such as cytokines and chemokines, which further attract and recruit other PMNs, monocytes, macrophages, and lymphocytes to the site of chronic inflammation. The bactericidal activity of neutrophils depends on cytoplasmic granules containing degradative lysosomal enzymes (Mortaz et al., 2018).

The blood vessel barrier is the main hindrance to the efficient and immediate transmigration of neutrophils to the site of inflammation. Therefore, it is highly needed to design a strategic nanotherapeutic approach to deliver nanoparticles such as; nanovesicles, metallic nanoparticles, protein nanoparticles, and polymer nanoparticles by using neutrophils as a delivery vehicle (Dong et al., 2017).

It has been documented that albumin nanoparticles can be incorporated into neutrophils and labeled with fluorescent dyes or radioactive agents for monitoring and analyzing the site of inflammation across the blood vessel barrier by using imaging techniques. (Chu et al., 2015).

It has also been reported that gold nanoparticles (Au NPs) were observed to be trapped by neutrophils in their extracellular traps (NETs). NETosis was found to be visible 15 min after AuNPs come in contact with neutrophils and trapped more NPs gradually. These NETs play a vital role in alerting the immune system to the signal of danger by the activation of TLR9, a DNA receptor. This activation starts the recruitment of neutrophils to the targeted site of inflammation (Yang et al., 2019).

This study will contribute with an advanced strategic nanotherapeutic approach to recruit activated neutrophils to the site of infection with maximum clearance and minimum toxicity.

#### 8.10.8 Interaction between nanoparticles and phagocytic cells (macrophages, dendritic cells)

Professional antigen-presenting cells (APCs), such as macrophages (Ms) and dendritic cells (DCs) are known as phagocytic cells that act as an interface between natural and acquired immunity. Ms is believed to be an efficient component of the innate immune system. They play role in the ingestion and killing of the pathogen in phagolysosomes by a mechanism called phagocytosis. They highly contribute to the rapid and non-specific removal of pathogens by reactive oxygen and nitrogen intermediates (Klopfleisch, 2016).

Antigen presentation is the main second function performed by Ms after phagocytosis. They process the pathogen after ingestion and present it in the form of antigen on the surface in association with class II MHC proteins along with costimulatory molecules such as CD86 and present it to the CD4 helper T-cells for adaptive immune response (Eiz-Vesper and Schmetzer, 2020).

Macrophages also synthesize and secrete several cytokines like IL-1 and Tumor necrosis factor (TNF), which play role in the training and activation of CD4 helper T-cells for the recognition and destruction of a pathogen to resolve different infections. Ms also secrete IL-8, a potent chemokine that attracts and recruit neutrophils and T-cells to the site of inflammation (Hughes et al., 2021).

The DCs are also regarded as professional APCs with the expression of Class II MHC protein on their surface. After phagocytosis of the pathogen, they process and present antigens in association with class II MHC protein to the helper T-cells for the stimulation of adaptive immunity. They also release several cytokines for the activation of natural killer cell (Yin et al., 2021).

Macrophages and dendritic cells are major phagocytes of the innate immune system with strong phagocytic ability. They capture nanoparticles loaded with therapeutic drugs (Ag) from blood circulation and accumulate them. The unique physiochemical properties of nanoparticles carry and protect Ag from degradative enzymes. These APCs are believed to be an ideal vehicle for the safe targeted delivery of drugs due to their efficient phagocytic ability (Banskota et al., 2017).

Several potential therapeutic strategies have been successfully developed using macrophages to deliver nanoparticles loaded with drugs efficiently (Lee et al., 2019; Xie et al., 2019). Drug-loaded nanoparticles can be prepared by loading chemotherapeutic drugs into nanoparticles for their safe arrival in the macrophages (Zhang et al., 2018; Hui et al., 2019). Different NPs such as lipid nanoparticles (LNPs), carbon nanotubes (CNTs), gold nanoparticles, and natural and artificially synthesized NPs have been used successfully to carry and deliver a drug to macrophages. Lipid nanoparticles (LNPs) have been used safely as drug delivery vessels (García-Pinel et al., 2019). The drug Patisiran can be delivered to macrophages by LNPs, which was approved by the FDA in 2018 (Hoy, 2018). The encapsulation of drugs by LNPs is a simple strategy, that not only protects macrophages from the side effects of drugs but also keeps them safe from carrier materials (Hou et al., 2021).

Multi-walled CNTs (MWCNTs) have also been reported as carrier nanoparticles of cancer antigens to be captured by DCs. The antigen was processed, released, and presented slowly on the surface of DCs in the association of MHC protein to activate CD helper T-cells and CD8 cytotoxic T-cells continuously for the regression of the tumor (Jia et al., 2018).

#### 8.10.9 Priming of mast cells with nanoparticles

Mast cells (MCs) are versatile effector cells of the body’s immune system with beneficial and deleterious effects against pathogens (Galli et al., 2020). They are abundantly found at the junctions between the tissues and the external environment such as skin and played a vital role in showing inflammatory response against pathogens by secreting inflammatory mediators after recognition (Marshall et al., 2019).

MCs originate from pluripotent stem cells in the bone marrow and mature with the development of secretory granules after reaching at tissue microenvironment by circulation. The activation and IgE-mediated degranulation of mast cells occur with the secretion of preformed inflammatory mediators such as histamine, heparin, lysosomal enzymes, and prostaglandins. MCs are an important first line of defense against various infectious agents due to the presence of TNF-α which recruit further neutrophils to the targeted site of infection and modulate both natural and acquired immune responses (Paivandy and Pejler, 2021).

Nanoparticles are believed to be novel modulators of mast cells for efficient response. Johnson and Duan have documented that nanoparticles can specifically target mast cells through FcεRI activation pathways. AgNPs possess antibacterial activity by stimulating mast cells through cell surface scavenger receptors. This results in the activation of intracellular signaling pathways and degranulation with the release of inflammatory mediators (Johnson et al., 2018). TiO2 nanoparticles enhanced the secretion of histamine and cytosolic Ca2+ concentration in mast cells without any prior exposure to an allergen (Duguay et al., 2020). Granules of mast cells have been reported to be a strong stimulator of adaptive immunity when they are degranulated at target sites of infection or vaccination (Jain et al., 2019). These findings may further explore the applications and utilizations of nanoparticles for diagnostic and therapeutic purposes of allergic diseases with the priming of mast cells, which could be of particular concern to allergic populations as the use of NPs in biomedical products are increasing rapidly.

#### 8.10.10 Therapeutic strategy of eosinophils for allergic issues

These leukocytes are characterized by the presence of orange or -red-stained granules in the cytoplasm and account for less than 3% of all leukocytes in human blood. They are originated and derived from CD34^+^ stem cells of bone marrow and released into blood circulation after maturation. The cytokines like interleukin (IL)-3, granulocyte-macrophage colony-stimulating factor (GMCSF), and IL-5 play a key role in the development and final differentiation of eosinophils respectively (Ramirez et al., 2018).

Eosinophils defend the body against parasitic, bacterial, viral, and fungal infections through mediators such as major basic protein (MBP), eosinophil peroxidase (EPO), eosinophil cationic protein (ECP), eosinophil-derived neurotoxin (EDN) in the presence of antibodies and complement (Kanda et al., 2020). The significance of eosinophils in inflammatory diseases of the skin, gastrointestinal and respiratory tract has been reported in the literature. The increased count of eosinophils in the blood or sputum sample of patients suffering from asthma is associated with the severity of the disease (Kanda et al., 2021).

NPS is believed to be a promising tool for the diagnosis and treatment of allergic diseases with the direct effect of NPs on eosinophils. It has been documented recently in the literature that AgNPs and ZnONPs have strong potential for the production of pro-inflammatory cytokine-like IL-8, which plays a significant role in the chemotaxis of neutrophils, basophils, and eosinophils to the target site (Vanharen and Girard, 2020). TiO_2_NPs recruit eosinophils from the blood circulation toward the inflamed area to fight against parasites and participate in immediate hypersensitivity by adhesion of eosinophils onto Endothelial Cells of blood vessels (Murphy-Marion and Girard, 2018).

This review may be helpful for researchers to understand the direct effects of various NPs on the biology and mechanism of action (MAO) of eosinophil cells. It will also be of great importance for better predicting their safer use in the diagnosis and treatment of hypersensitivity reactions.

#### 8.10.11 Anti-allergic role of basophils along with NPs inhibitor conjugates

Basophils are granular leukocytes with blue-stained cytoplasmic granules. They are the least abundant circulating granulocytes which account for less than 1% of all leukocytes. They resemble mast cells due to the expression of the high-affinity immunoglobulin E (IgE) receptor (FcεRI). Basophils act as gatekeepers to control the intrusion of eosinophils to the target site with the release of Th2-related cytokines like IL-4 and IL-13 after binding of FcεRI with IgE due to exposure of allergen. The activated basophils increase vascular permeability and mucus production due to the secretion of histamine and LTC_4_ (Iype and Fux, 2021).

The conjugate of allergen and gold nanoparticles AuNPs effectively and vigorously stimulate basophils and cause degranulation with the secretion of preformed mediators including histamine, prostaglandins, leukotrienes, and proteases to mediate immediate and delayed inflammatory immune response (Radauer-Preiml et al., 2015).

Gold nanoparticles (AuNPs) can successfully be used to target and inhibit the IgE-dependent degranulation of basophils with signal transduction inhibitors such as calcineurin and anti-CD203c. AuNPs are relatively non-toxic anti-inflammatory nanoparticles that can be conjugated with pharmacological agents to stimulate anti-allergic responses (Yasinska et al., 2019). The specific targeting of basophils with gold nanoconjugates and signal transduction inhibitors indicates that this technology could be used as a therapeutic potential treatment for allergic diseases with minimal side effects.

#### 8.10.12 Impact of nanoparticles on the stimulation of adaptive immunity

Adaptive immunity is also known as acquired immunity, a specific second-line long-term defense mediated by B and T cells which enables the host to develop a rapid response upon second exposure to antigen. The cells of adaptive immunity require APCs for the recognition of antigens. APCs phagocytose, process, and present antigens to T-cells in association with MHC protein. Nanoparticles target and interact with circulating APCs for efficient response (Marshall et al., 2018).

##### 8.10.12.1 Consequences of nanoparticles on T cells

Nanotechnological techniques are believed to be a potential therapeutic strategy for the effective treatment of many diseases by specifically targeting the region without any detrimental effects. Drug delivery systems based on nanoparticles can be made highly efficient by considering the different physical and chemical properties of nanoparticles such as; size, shape, charge, stability, *etc.*

Superparamagnetic iron oxide nanoparticles (SPIONPs) target and accumulate T cells in the specific region of interest with the application of an external magnetic field (EMF). This strategy has been proven beneficial in cancer treatment and vaccine preparation (Day et al., 2021). Poliglusam nanoparticles (Polymeric nanoparticles) possess the potential to reduce the size of tumors, particularly of breast origin safely by activating host immunity. It has been documented that Poliglusam has an intrinsic inclination in increasing the production of IFN-γ by activated T lymphocytes in cancer cells. These immunotherapeutic effects could be made more effective along with the use of anti-cancerous medication for the complete removal of breast cancer (Soleimani et al., 2019).

Synthetic Janus nanoparticles have been reported to be used in adoptive cancer immunotherapy. These nanoparticles with clustered ligands on the surface stimulate T cells with an inadequate amount of stimulus (Lee and Yu, 2017). Nanoparticles can also be used successfully in the treatment of brain tumors by crossing the blood-brain barrier (BBB) and enhancing T-cell adoptive therapy. The activation of T-cells highly depends upon the size and high surface-to-volume ratio of carbon nanotubes which plays role in the encapsulation of antigens or cytokines (Balakrishnan and Sweeney, 2021).

The vaccine is a strong and economical approach to defending the body against infections by limiting pathogenesis and spreading of disease with the advanced utilization of nanoparticles. Inorganic carboxylated polystyrene nanoparticles stimulate B-cells, helper T-cells, and cytotoxic T-cells to provoke humoral and cell-mediated responses respectively. Fluorescent nanoparticles can be used to trace the attachment of these nanoparticles with cell subsets specifically by flow cytometry (Wilson et al., 2020). Metal-based nanoparticles have also been documented to stimulate T cells for immune responses. TiO2 nanoparticles play role in the activation and expansion of T cells with the release of inflammatory cytokines. Gold nanoparticles can polarize Th2 and Th17 cells. The induction of Th1 or Th2 immune response decision is dependent on the size of nanoparticles after the introduction of antigen and Nanoparticle conjugate in the body (Luo et al., 2015). This review will guide and explore new ways to develop vaccines and immunotherapies to treat cancer and combat other viral diseases by activating T-cells using a nanotechnological approach.

## 9 Effects of nanoparticles on B cells

B lymphocytes are another important component of the adaptive immune system which combat bacterial diseases by the production of antibodies. They act as APC and present surface IgM receptors for the recognition and attachment of antigens. They internalize, process, and present antigen on its surface in conjunction with MHC class II protein after exposure. B lymphocytes present this complex of antigen and MHC II to T-cell receptors (TCR) of CD4 cells. The T-cells activate and differentiate these B-cells into Ab-producing plasma cells with the production of various interleukins such as; IL-2, IL-4, and IL-5 (Van Langelaar et al., 2020).

The humoral immune response can be achieved enhancedby the use of a nanotechnological approach. It has been documented that antigens are released slowly from the depot form of nano vaccines at a predetermined rate to provoke B-cells for the production of antibody-mediated responses against infections (Singh, 2021). TiO_2_ nanoparticles are believed to have the potential to elicit Ab-mediated response by B-cells due to increased levels of IL-4 by activated T-cells with minimal toxicity (Malachowski and Hassel, 2020).

The combination of calcium phosphate nanoparticles (CaPNs) and antigenic moiety stimulate B-cells to increase the level of antibodies with a unique circular shape and smooth surface. They are considered a promising candidate for the development of a novel vaccine to elicit a humoral immune response against *B. melitensis and B. abortus* (Sadeghi et al., 2020)*.* Iron oxide nanoparticles (IONPs) are widely used nanoparticles in various medical applications due to unique characteristics like high surface-to-volume ratio and super magnetism. They are particularly important in generating humoral immune responses by B-cells as compared to cell-mediated responses by T-cells (Gaharwar et al., 2020).

This latest review data from the literature may help design advanced theragnostic applications by using different combinations of nanoparticles and antigens.

### Other applications of nanobiotechnology

Some other applications of nanobiotechnology include the preparation of hydrogel biomaterials which show promising regenerative effects for damaged corneal tissues (Khosravimelal et al., 2021a). Moreover, it can also help us prepare fabricated antimicrobial nanofibers. These nanofibers show increased antimicrobial activity and can be used as a wound dressing to decrease infection and enhance healing properties (Khosravimelal et al., 2021b). Additionally, Nanopatterned surfaces have shown promising results in stem cell differentiation. However, there is a need for more research on this topic (Eftekhari et al., 2021). Apart from their significance in regenerative medicine, antimicrobials, drug delivery, and stem biology, Nanobiomaterials can be used for the treatment of diabetes mellitus 1 by generating supplemental oxygen required by islets until the formation of blood vessels (Gholipourmalekabadi et al., 2016). Electrospinning uses the principles of nanobiotechnology for the preparation of biocompatible scaffolds which mimic collagen nanofibers providing protection and mechanical support at the injury site (Farshi et al., 2022).

## 10 Conclusion and future perspectives

Nanotechnological techniques can be used to develop and design cargo systems for the delivery of vaccines, depot formulations, adjuvants, and drugs in association with various types of nanoparticles to trigger cell-mediated and humoral immunological responses by T-cells and B-cells respectively to combat different viral and bacterial infections. Different types of nanoparticles such as organic, inorganic, metallic, non-metallic, and polymeric NPs are believed potentially strong to be widely used in medical applications, such as diagnostic purposes, therapeutic strategies, and gene and drug delivery to the targeted area of interest.

The interactions of nanoparticles and Ag with the immune system have gained attention due to the strong stimulation of immune cells to mediate cell-mediated and humoral immunity in cancer and viral diseases. Although nanoparticles are becoming a useful candidates in various advanced theragnostic applications still, we are facing many health issues due to limited explored knowledge of nanoparticles.

Comprehensive studies are highly needed to explore further advanced pharmacodynamics, pharmacokinetics, immunomodulatory, and toxicity effects related to different types of nanoparticles critically affecting the health of human beings. A collaboration between nanotechnology and immunology is an emerging field of interest with a strong potential to design diagnostic and therapeutic applications to control health issues (Table 2) (Taniguchi, 1974; Mansoori and Soelaiman, 2005; Feynman, 2018).

**TABLE 2:** Some important applications of nanotechnology.

Nanoparticle type	Size (nm)	Administration type	Mechanism of action	Target/Disease	References
Liposome	<100	Intravenous	Drug delivery	Parkinson	Saeedi et al. (2019)
Carbon Nanotubes	3.5–7	Intraperitoneal	Endocytosis	Parkinson	Saeedi et al. (2019)
Polymeric NPs	1–100	Oral	Drug delivery	Diabetes Mellites	Simon-Deckers et al. (2009)
Layered Double Galactose	40–300	Directly exposed to cells (in the lab)	Target drug delivery	Hepatocellular Carcinoma	Sadeghi et al. (2020)
Al2O3	60	Direct on surface (in Lab)	particle penetration	*E. coli, B. subtilis, Pseudomonas*	Jiang et al. (2009)
Silver	50	Direct on the surface (in the lab)	Membrane disruption, Interferes with replication	*E. coli*	Pal et al. (2007)
Hydrogels	370–800	3D scaffold	Mimic the function of specific cell layers	Cornea wound healing	Khosravimelal et al. (2021a)
Nano-Ag	5–20	Direct on surface	strong and wide-spectrum antimicrobial	Contaminated Water	Qu et al. (2013)
diatomite	10–200 μm	Direct exposure to cells (in the lab)	siRNA delivery	Cancer	Rea et al. (2014)
mesoporous silica	200	Nanocarrier	gene delivery applications	Hela cells and macrophages	Park et al. (2008)
